# Increased *Trypanosoma* spp. richness and prevalence of haemoparasite co-infection following translocation

**DOI:** 10.1186/s13071-019-3370-6

**Published:** 2019-03-21

**Authors:** Amy S. Northover, Stephanie S. Godfrey, Sarah Keatley, Alan J. Lymbery, Adrian F. Wayne, Crystal Cooper, Louise Pallant, Keith Morris, R. C. Andrew Thompson

**Affiliations:** 1 College of Science, Health, Engineering and Education, Murdoch University, 90 South Street, Murdoch, Western Australia 6150 Australia; 2 Department of Zoology, University of Otago, 362 Leith Street, Dunedin, 9016 New Zealand; 3 Centre for Sustainable Aquatic Ecosystems, Harry Butler Institute, Murdoch University, 90 South Street, Murdoch, Western Australia 6150 Australia; 4 Biodiversity and Conservation Science, Department of Biodiversity, Conservation and Attractions, Brain Street, Manjimup, Western Australia 6258 Australia; 5 Biodiversity and Conservation Science, Department of Biodiversity, Conservation and Attractions, Wildlife Place, Woodvale, Western Australia 6946 Australia

**Keywords:** *Babesia*, *Bettongia penicillata*, Fauna translocation, Piroplasm, Polyparasitism, *Theileria*, Trypanosome

## Abstract

**Background:**

Understanding how fauna translocation and antiparasitic drug treatment impact parasite community structure within a host is vital for optimising translocation outcomes. *Trypanosoma* spp. and piroplasms (*Babesia* and *Theileria* spp.) are known to infect Australian marsupials, including the woylie (*Bettongia penicillata*). However relatively little is known about these haemoparasites, or how they respond to management practices such as translocation. We monitored haemoparasites infecting woylies for up to 12 months during two fauna translocations to supplement existing woylie populations in three different sites (Dryandra, Walcott and Warrup East) within south-western Australia between 2014 and 2016, with the aim of investigating (i) how haemoparasite prevalence, *Trypanosoma* spp. richness and *Trypanosoma* spp. community composition varied over time and between different sites following translocation; and (ii) whether ivermectin treatment indirectly impacts haemoparasite prevalence. Using molecular methods, 1211 blood samples were screened for the presence of trypanosomes, and a subset of these samples (*n* = 264) were also tested for piroplasms.

**Results:**

Trypanosomes and piroplasms were identified in 55% and 94% of blood samples, respectively. We identified five *Trypanosoma* species, two *Theileria* species, a single species of *Babesia* and a novel *Bodo* species. *Trypanosoma* spp. richness and the prevalence of haemoparasite co-infection increased after translocation. Prior to translocation, *Trypanosoma* spp. community composition differed significantly between translocated and resident woylies within Walcott and Warrup East, but not Dryandra. Six months later, there was a significant difference between translocated and resident woylies within Dryandra, but not Walcott or Warrup East. The response of haemoparasites to translocation was highly site-specific, with predominant changes to the haemoparasite community in translocated woylies occurring within the first few months following translocation. Ivermectin treatment had no significant effect on haemoparasite prevalence.

**Conclusions:**

This study contributes to our understanding of haemoparasite dynamics in woylies following translocation. The highly site-specific and rapid response of haemoparasites to translocation highlights the need to better understand what drives these effects. Given that haemoparasite prevalence and composition of translocated and resident animals changed significantly following translocation, we propose that parasite monitoring should form an essential component of translocation protocols, and such protocols should endeavour to monitor translocated hosts and cohabiting species.

**Electronic supplementary material:**

The online version of this article (10.1186/s13071-019-3370-6) contains supplementary material, which is available to authorized users.

## Background

Suitably termed a “biological package”, a host and its suite of parasites are unique, coexisting but continually adapting entities, enhancing the competitive fitness and ultimately affecting the survival of one another [1, 2]. While it is now recognised that polyparasitism (co-infection, concomitant infection or multiparasitism) is the rule rather than the exception in wildlife [3, 4], deciphering the manner in which parasites interact with each other and their host is complex; even more so in situations where perturbations to the host-parasite community are likely to occur.

Fauna translocations play a pivotal role in the management of threatened species worldwide. However, the act of moving a host and its infracommunity of parasites from one ecosystem to another will inevitably disrupt host-parasite associations [2, 5, 6]. Changes in the composition of the parasite community may significantly impact host health and population dynamics [7]. Likewise, translocation-associated stress may enhance susceptibility to parasite infection [8], or promote recrudescence of latent disease [9]. Unfortunately, translocation protocols rarely incorporate parasite monitoring and field studies that examine parasite prevalence pre- and post-translocation, e.g. [10] are rare; thus we lack an understanding of how translocation impacts the host-parasite community [11]. Additionally, antiparasitic drugs may be administered to a host, often without any attempt to evaluate treatment efficacy [12]. With demonstrated effects of antiparasitic drugs in target and non-target parasites, e.g. [13], there is the potential to negatively impact translocation outcomes.

The critically endangered woylie (syn. brush-tailed bettong *Bettongia penicillata*) is a small macropodid marsupial, which once occupied most of southern Australia. Adult woylies weigh between 1.0–1.6 kg, measure roughly 600 mm in length (nose to tail tip) and live for approximately 4–6 years in the wild [14]. Over the past decade, woylies have undergone greater than 90% population declines and are now restricted to three remaining wild indigenous populations (Kingston, Perup and Dryandra) in south-western Australia [15]. Both the Kingston and Perup woylie populations are located within the Upper Warren region, where woylie population declines were most pronounced [16]. While various hypotheses have been proposed to explain the declines, their spatio-temporal pattern suggests the potential role of an infectious disease agent [15]. Woylie monitoring carried out immediately prior to, and during the declines, detected a high prevalence of skin disease, but despite investigation, a causative disease agent could not be found [16]. Since then, the focus of investigation has shifted toward the potential role of other disease agents and *Trypanosoma* spp. have been of particular interest [17–20].

Five *Trypanosoma* species have been detected in woylies: *T. copemani*, *T. vegrandis* [18], *T. noyesi* [21], *Trypanosoma* sp. ANU2 and *T. gilletti* [22]. Two distinct genotypes of *T. copemani* are formally recognised, *T. copemani* genotype 1 (G1) and *T. copemani* genotype 2 (G2) [18]. *Trypanosoma copemani* G2 has been detected in woylie tissues by PCR, associated with tissue pathology, and is capable of invading cells *in vitro* [18, 23]. Molecular studies have identified a higher prevalence of *T. copemani* and *Trypanosoma* spp. co-infection in a declining woylie population compared to a stable population [17–19], and the extent of *Trypanosoma* co-infection was found to increase during the decline [20].

Various piroplasms (*Theileria* and *Babesia* spp.) have also been identified in Australian wildlife, including *Theileria penicillata* [24] and a *Babesia* sp. [25] in woylies. While piroplasms have been associated with significant clinical disease in domestic livestock and companion animals [26], relatively little is known about their biology, transmission and clinical impact within native Australian wildlife [27]. To date, *T. penicillata* has been detected at high prevalence (> 80%) in several woylie populations [28], while *Babesia* sp. infection has been found at moderate prevalence (47%) in a single wild woylie population [25].

As part of ongoing management of the woylie, remaining wild populations are periodically supplemented by translocations [15]. Like many threatened wildlife species, parasite monitoring is not routinely undertaken and there is little understanding of how translocations impact parasite community structure in woylies. In this longitudinal field-based study, we examined *Trypanosoma* spp. and piroplasms (*Theileria* and *Babesia* spp.) infecting woylies during two translocations to supplement existing woylie populations. Specifically, we aimed to investigate (i) how haemoparasite prevalence, *Trypanosoma* spp. richness and *Trypanosoma* spp. community composition varied over time and between different sites following translocation; and (ii) whether ivermectin treatment indirectly affects haemoparasite prevalence. We predicted that haemoparasite prevalence would decrease following translocation, in a similar manner to fauna reintroductions, where parasite loss commonly occurs [29, 30]. As ivermectin targets nematodes and arthropods, we did not expect ivermectin to directly affect *Trypanosoma* spp. prevalence in woylies, but we predicted that the impact of ivermectin on arthropod vectors may indirectly reduce trypanosome transmission.

## Methods

### Study sites and trapping regime

**Fig. 1:**
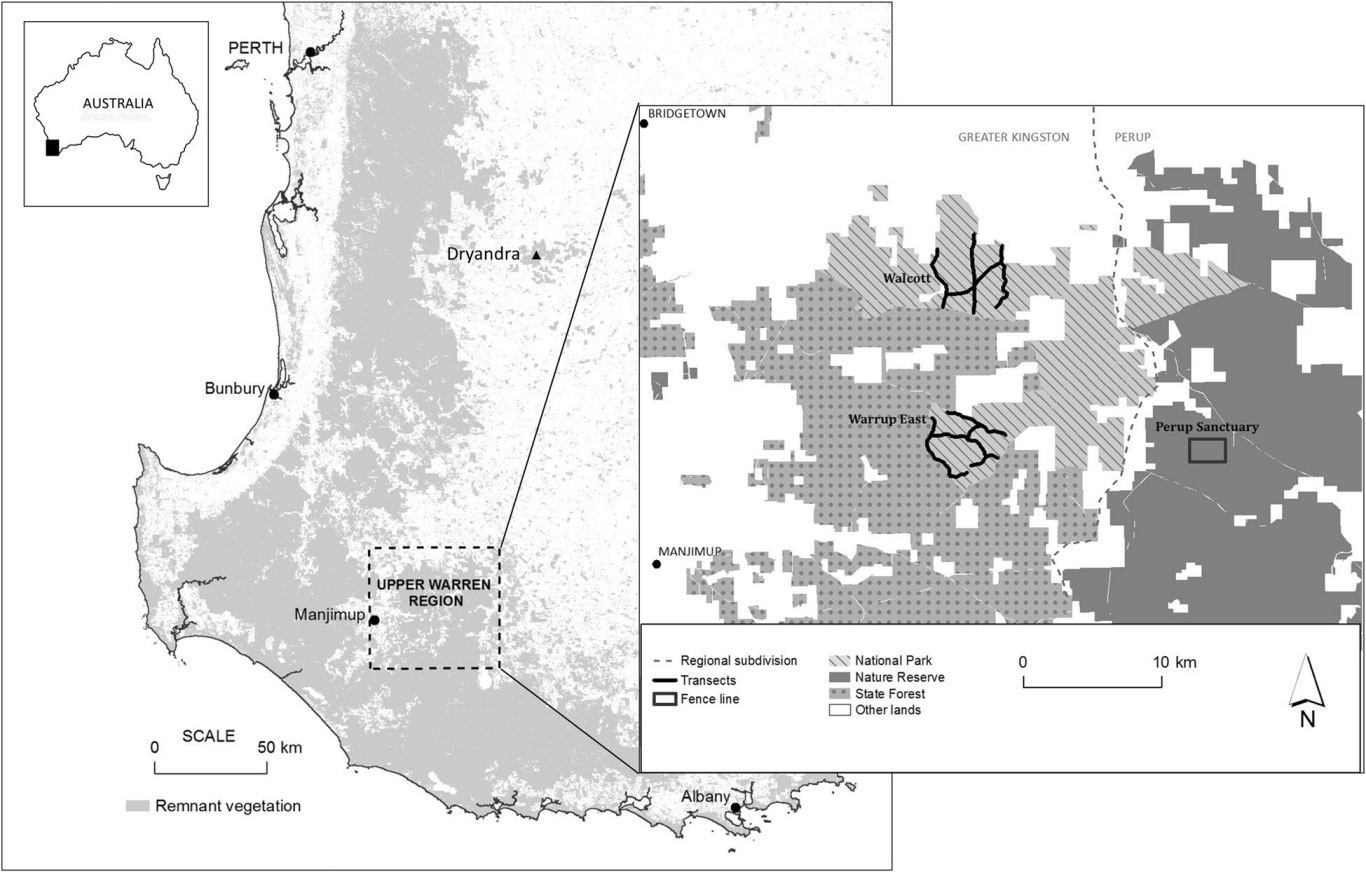
Map depicting the study sites within the south-west of Western Australia. The box (right), shows Walcott and Warrup East in relation to Perup Sanctuary. Dryandra is located approximately 250 km north-east of the Upper Warren region

During the first translocation (June 2014), 182 woylies were translocated from Perup Sanctuary, a 423-ha fenced reserve situated within the Tone-Perup Nature Reserve near Manjimup in Western Australia (34.2506°S, 116.1425°E), to supplement two unfenced wild populations [Walcott (*n* = 92) and Warrup East (*n* = 90)] within the Upper Warren region (Fig. 1). Much of the native forests within this region are dominated by jarrah (*Eucalyptus marginata*), marri (*Corymbia callophyla*), and occasionally wandoo (*Eucalyptus wandoo*) [15]. During the translocation, woylies (equal ratio of male to female) were captured over three consecutive nights using Sheffield cage traps (Sheffield Wire Products, Welshpool, Western Australia) set along multiple transects (≤ 280 traps/night, 100 m spacing) at dusk, baited with universal bait (rolled oats, peanut butter and sardines), and cleared within three hours of sunrise the following day. Monitoring was carried out over three-four consecutive nights at one (July), three (September), six (December), ten (April 2015) and eleven (May 2015) months after translocation. All post-translocation trapping except May 2015 (grid trapping; 7 × 7 traps, 50 m apart) consisted of transect trapping (≤ 106 traps/night, 100–200 m spacing). Resident woylies within each destination site were also sampled prior to translocation (April and May 2014), using a grid (as above) and transect trapping (≤ 100 traps/night, 100–200 m spacing).

The second translocation (June 2015) involved the relocation of 69 woylies (47 male, 22 female) from various wild sites within the Upper Warren, into an unfenced site within Dryandra woodland (32.8027°S, 116.8854°E). Dryandra is situated in the Western Australian wheatbelt, roughly 250 km north-east of the Upper Warren (Fig. 1). The open-canopy woodlands in this region are dominated by wandoo, powderbark wandoo (*Eucalyptus accedens*), brown mallet (*Eucalyptus astringens*) and to a lesser extent marri [31]. During the translocation, woylies were captured (as above) over four consecutive nights. Monitoring was carried out over four consecutive nights at one (July), two (August), three (September), six (December), nine (March 2016) and twelve (June 2016) months following translocation. All post-release trapping except June 2016 (grid trapping, as above) consisted of transect trapping (100 traps/night, 200 m spacing). Resident woylies within the release site were also sampled prior to translocation (June 2015) using a grid and transects (as above).

### Woylie identification and ivermectin treatment

Each woylie was identified with two uniquely numbered ear-tags. Prior to translocation, half of the woylies [Dryandra (*n* = 35); Walcott (*n* = 47); and Warrup East (*n* = 46)] were administered a single subcutaneous dose of ivermectin (Ivomec® 0.2 mg/kg). While the ratio of treated male to female woylies was equal within the Upper Warren, there were a greater number of treated males (24/35) than females (11/35) within Dryandra.

### Haemoparasite detection

Blood was collected from the lateral tail vein into MiniCollect® EDTA tubes (Greiner Bio-One, Frickenhausen, Germany) and frozen at -20 °C prior to processing. DNA was extracted from 200 µl aliquots of whole blood using the QIAmp 96 DNA blood kit as per the manufacturer’s instructions (Qiagen, Hilden, Germany), with a final elution volume of 60 µl. A negative control was included in the extraction process.

DNA was screened for the presence of trypanosomes using a nested set of generic trypanosome primers (Additional file 1: Table S1) designed by [32] and [33], which target the second fragment of the conserved *18S* rDNA gene region. Samples that tested positive for trypanosomes were subsequently screened for the presence of *T. copemani*, *T. vegrandis* and *T. noyesi* using specific nested primers designed by [18] and [33] (Additional file 1: Table S1). Although *T. gilletti* is described as a distinct species [33], it is very closely related to *T. vegrandis* [22], and species-specific PCR primers for *T. vegrandis* are unable to discriminate between the two species; thus *T. gilletti* could only be identified by DNA sequencing (see below). All PCR reactions were performed as described by [22], with the exception that 2 μl of DNA was added to a 24 μl master mix.

A nested PCR was used to screen for the presence of piroplasms using primers (Additional file 1: Table S1) and conditions (Additional file 1: Table S2) modified from [34]. Negative and positive controls were included in all PCR reactions, with the positive control derived from a known positive stock. Haemoparasites were identified based on expected band size for each species (Additional file 1: Table S1). PCR products were purified using either the Agencourt AMPure PCR purification system, or by using an in-house filter tip method described by [35].

Samples that tested positive for trypanosomes using the generic primer set, but negative for specific *Trypanosoma* species using clade-specific primers underwent Sanger sequencing at the *18S* rDNA locus (Additional file 1: Table S2). All piroplasm-positive samples were sequenced. Phylogenetic analyses (Additional file 1: Table S2) were conducted for putative new haemoparasites (see “Results”; Additional file 2: Figure S1, Additional file 3: Figure S2, Additional file 4: Figure S3).

### Data analysis

For each haemoparasite species, prevalence of infection was calculated as the proportion of infected individuals with Jeffrey’s 95% confidence intervals (CI) calculated assuming a binomial distribution. We estimated trypanosome infracommunity richness (polyparasitism) in terms of the number of *Trypanosoma* spp. (*T. copemani*, *T. vegrandis*, *T. noyesi*, *Trypanosoma* sp. ANU2 and *T. gilletti*) infecting a host. Piroplasms were excluded from our analyses of parasite infracommunity richness and community composition (see below) as piroplasm infection was only assessed in a subset of translocated woylies (*n* = 264) between June and September. To evaluate the impact of site (Dryandra, Walcott and Warrup East), time since translocation (TST) and ivermectin treatment (translocated woylies only), and their interactions, on the presence of each haemoparasite species, and on *Trypanosoma* spp. richness, we used generalised linear mixed-effects models (package *lme4*, [36]) in R (version 6.1.15; [37]). We could not run these models for *T. noyesi*, *Trypanosoma* sp. ANU2, *T. gilletti*, *Th. apogeana* genotype ANO2 or *Babesia* sp., as too few individuals were infected (prevalence 7.5%, 2.3%, 0.6%, 16.3% and 4.5%, respectively). All analyses were conducted separately for translocated and resident woylies. For each of our models, we tested for collinearity using variance inflation factors, and residuals were checked for normality/outliers to ensure model validity. Presence/absence data for all haemoparasites were modelled as binomial variables with a logit link function. Measures of *Trypanosoma* spp. richness were modelled as Poisson variables with a log link function. To account for repeated measures of individuals after translocation, woylie ID was included as a random effect.

Differences in *Trypanosoma* spp. community composition (presence/absence data only) between translocated and resident woylies were evaluated twice for each site; pre-translocation and 6 months after translocation. For each site at each time point, dissimilarities in community composition among hosts were estimated from presence/absence data with the Bray-Curtis coefficient. Differences in community composition between groups were visualised with non-metric multidimensional scaling plots, with the variance in mean rank order of similarity values within and between groups (R) tested for significance by a permutation technique applied to the pairwise dissimilarity matrix (one-way ANOSIM, implemented in PRIMER v. 6.0; [38]). The contribution of individual *Trypanosoma* spp. to differences in composition among host groups was assessed by averaging the Bray-Curtis coefficients for each species over all pairwise host combinations, using the SIMPER procedure in PRIMER.

## Results

We analysed 1211 blood samples collected from 631 individual woylies (380 residents, 251 translocated) for the presence of haemoparasites (Additional file 1: Tables S3-S5). Of these, 49 samples could not be identified to the species level for any of the haemoparasites, so they were excluded from our analyses and prevalence estimates. Pre-translocation woylie capture rates (total number of captures divided by the total trap effort; [39]) were much higher within Walcott (0.11) compared to Warrup East (0.05) or Dryandra (0.04). DNA sequencing revealed the presence of three novel haemoparasites, which are referred to here as *Trypanosoma* sp. ANU2 (MF459652; a putative new species genetically characterised by [22]), *Theileria apogeana* genotype ANO2 (MK182522), and *Bodo* sp. ANO4 (MK182523). For each novel haemoparasite, genetic similarity was compared to other previously described species (Additional file 1: Table S6). *Trypanosoma gilletti* was also identified in woylies for the first time [22].

Overall, trypanosomes were detected in 55.3% of samples. Of the 1162 samples that could be identified to species (or putative species), *T. vegrandis* was the most common (32.5%), followed by *T. copemani* (23.9%), *T. noyesi* (7.5%), *Trypanosoma* sp. ANU2 (2.3%) and *T. gilletti* (0.6%). *Trypanosoma* sp. ANU2 and *T. gilletti* were only found in woylies originating from, or trapped within, the Upper Warren region (Additional file 1: Tables S3-S5). Piroplasms were highly prevalent (93.6%) in the subset of samples we tested (translocated woylies only). *Theileria penicillata* was identified in 73.5% of samples, while *Th. apogeana* genotype ANO2 (16.3%) and *Babesia* sp. (4.5%) were comparatively rare. *Theileria apogeana* genotype ANO2 was common in translocated woylies originating from Perup Sanctuary, but rare outside of the reserve (Additional file 1: Tables S3-S5). *Babesia* sp. was infrequently detected in woylies originating from Perup Sanctuary (*n* = 1, Walcott) and was predominantly isolated in woylies obtained from wild Upper Warren sites (Additional file 1: Table S3). A *Bodo* species (*Bodo* sp. ANO4) was detected in a single woylie from Perup Sanctuary.

### Effect of site on haemoparasite prevalence

**Fig. 2:**
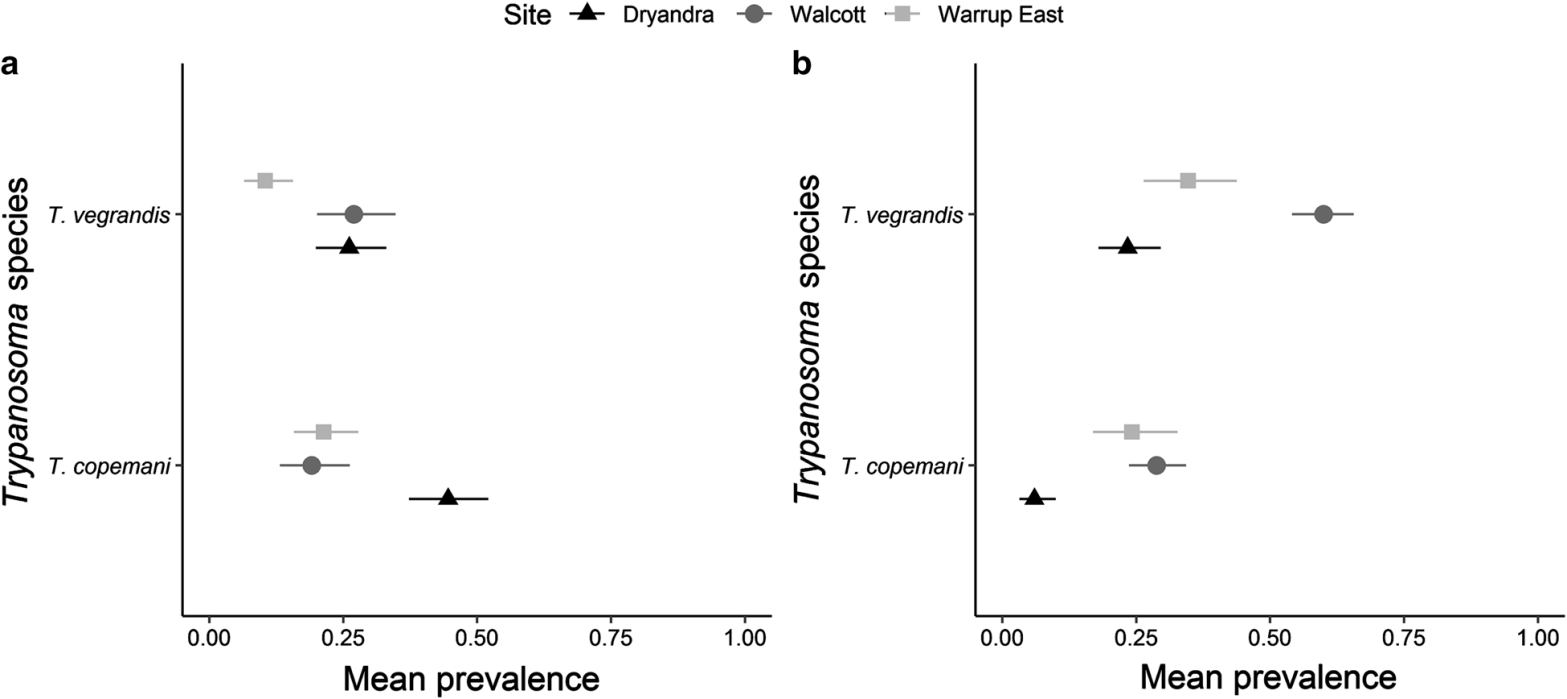
The overall impact of site on *Trypanosoma* spp. prevalence (with 95% CI) in translocated (**a**) and resident (**b**) woylies

In translocated and resident woylies, both *T. copemani* and *T. vegrandis* prevalence differed significantly between sites (Table 1). In translocated woylies, *T. copemani* prevalence was markedly higher in Dryandra than in Walcott or Warrup East, whereas *T. vegrandis* prevalence was much lower in Warrup East than the other sites (Fig. 2a). In resident woylies, both *T. copemani* and *T. vegrandis* prevalence were on average highest within Walcott (particularly *T. vegrandis*) and lowest within Dryandra (Fig. 2b). There were no differences in *Th. penicillata* prevalence in translocated hosts among sites (Table 1).

**Table 1:** Results from generalised linear mixed model analysis of factors influencing haemoparasite prevalence and *Trypanosoma* spp. polyparasitism in translocated and resident woylies

	*Trypanosoma copemani*			*Trypanosoma vegrandis*			*Trypanosoma* spp. polyparasitism			*Theileria penicillata*		
	*χ* ^2^	*df*	*P*	*χ* ^2^	*df*	*P*	*χ* ^2^	*df*	*P*	*χ* ^2^	*df*	*P*
Translocated												
Site	16.621	2	**<** **0.001**	14.842	2	**0.001**	10.420	2	**0.005**	2.923	2	0.232
TST (time since translocation)	4.934	1	**0.026**	28.180	1	**<** **0.001**	17.054	1	**<** **0.001**	2.462	1	0.117
Ivermectin	0.186	1	0.666	0.632	1	0.426	0.063	1	0.802	1.595	1	0.207
Site:TST	2.685	2	0.261	16.827	2	**<** **0.001**	3.952	2	0.139	9.611	2	**0.008**
Site:Ivermectin	3.557	2	0.169	5.340	2	0.069	8.950	2	**0.011**	1.225	2	0.542
TST:Ivermectin	0.005	1	0.946	3.944	1	**0.047**	1.150	1	0.284	0.006	1	0.938
Site:TST:Ivermectin	0.657	2	0.720	1.924	2	0.382	0.067	2	0.967	–	–	–
Resident												
Site	29.158	2	**<** **0.001**	43.527	2	**<** **0.001**	65.988	2	**<** **0.001**	–	–	–
TST	6.487	1	**0.011**	0.933	1	0.334	0.006	1	0.940	–	–	–
Site:TST	2.744	2	0.254	2.757	2	0.252	1.740	2	0.419	–	–	–

Significant (*P* < 0.05) results are highlighted in bold

### Effect of time since translocation on haemoparasite prevalence

**Fig. 3:**
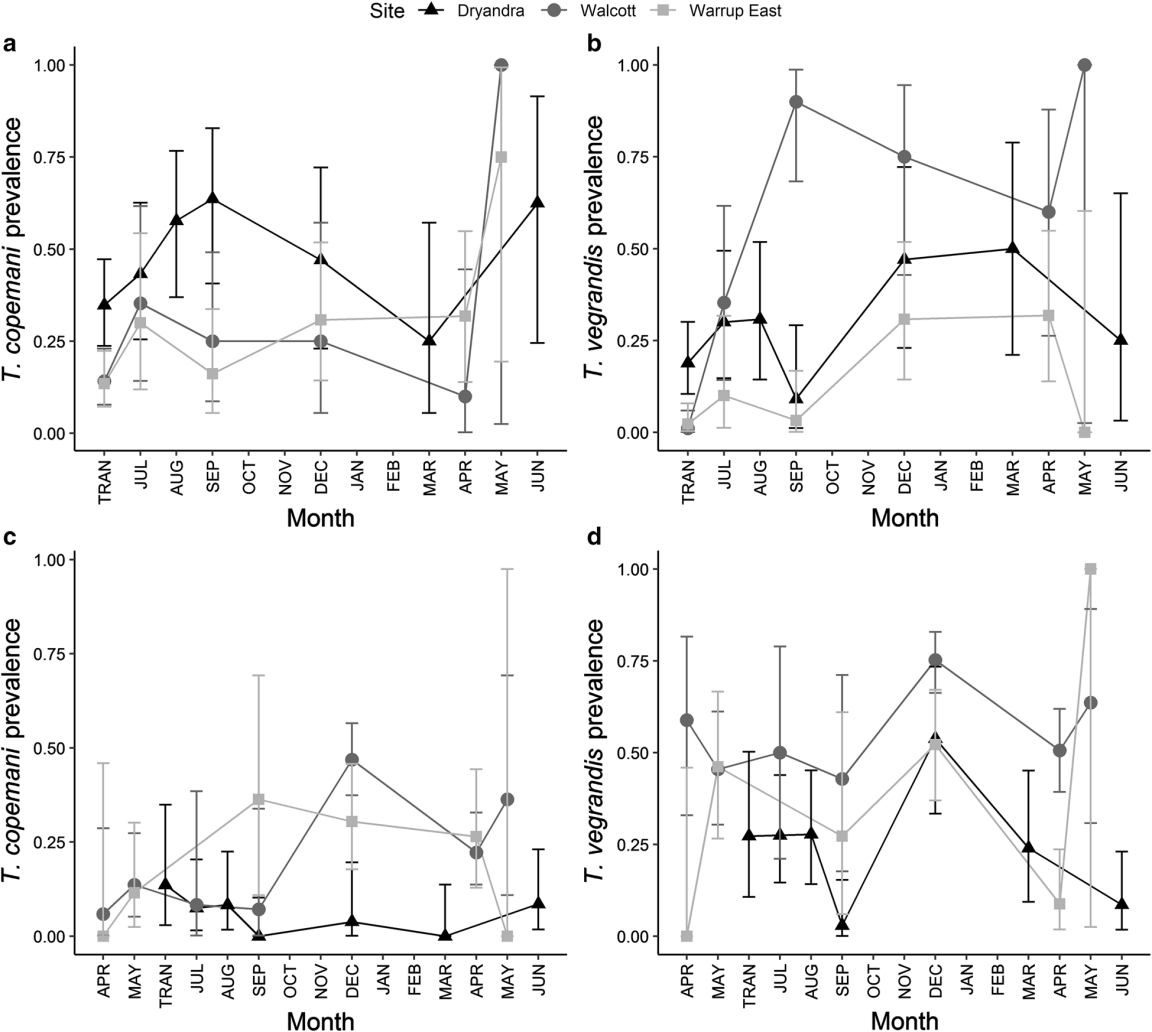
*Trypanosoma* spp. prevalence over time (with 95% CI) in translocated (**a**, **b**) and resident (**c**, **d**) woylies. TRAN: time of translocation

In translocated woylies, *T. copemani* prevalence increased in all sites with TST, while *T. vegrandis* prevalence increased in all sites except Warrup East, leading to a significant interaction between TST and site for this species (Table 1, Fig. 3a, b). In resident woylies, the prevalence of *T. copemani*, but not *T. vegrandis*, decreased with TST (Table 1, Fig. 3c, d).

**Fig. 4:**
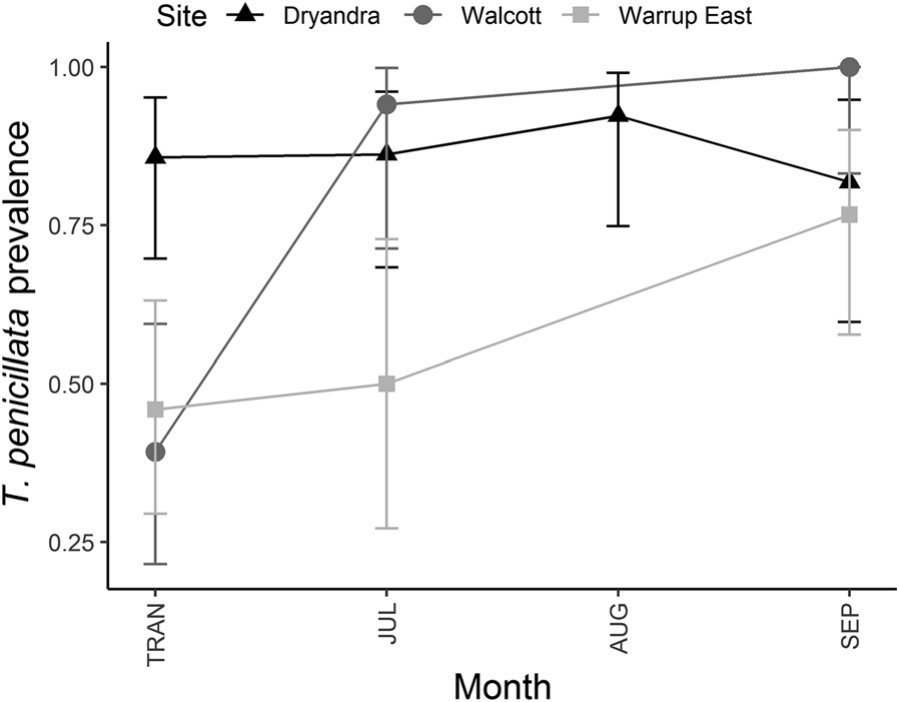
*Theileria penicillata* prevalence over time (with 95% CI) in translocated woylies. TRAN: time of translocation

In translocated woylies, there was a significant interaction between TST and site for *Th. penicillata* (Table 1), with prevalence increasing over time in Walcott and Warrup East, while remaining high within Dryandra (Fig. 4).

### Haemoparasite community structure in translocated and resident woylies

**Fig. 5:**
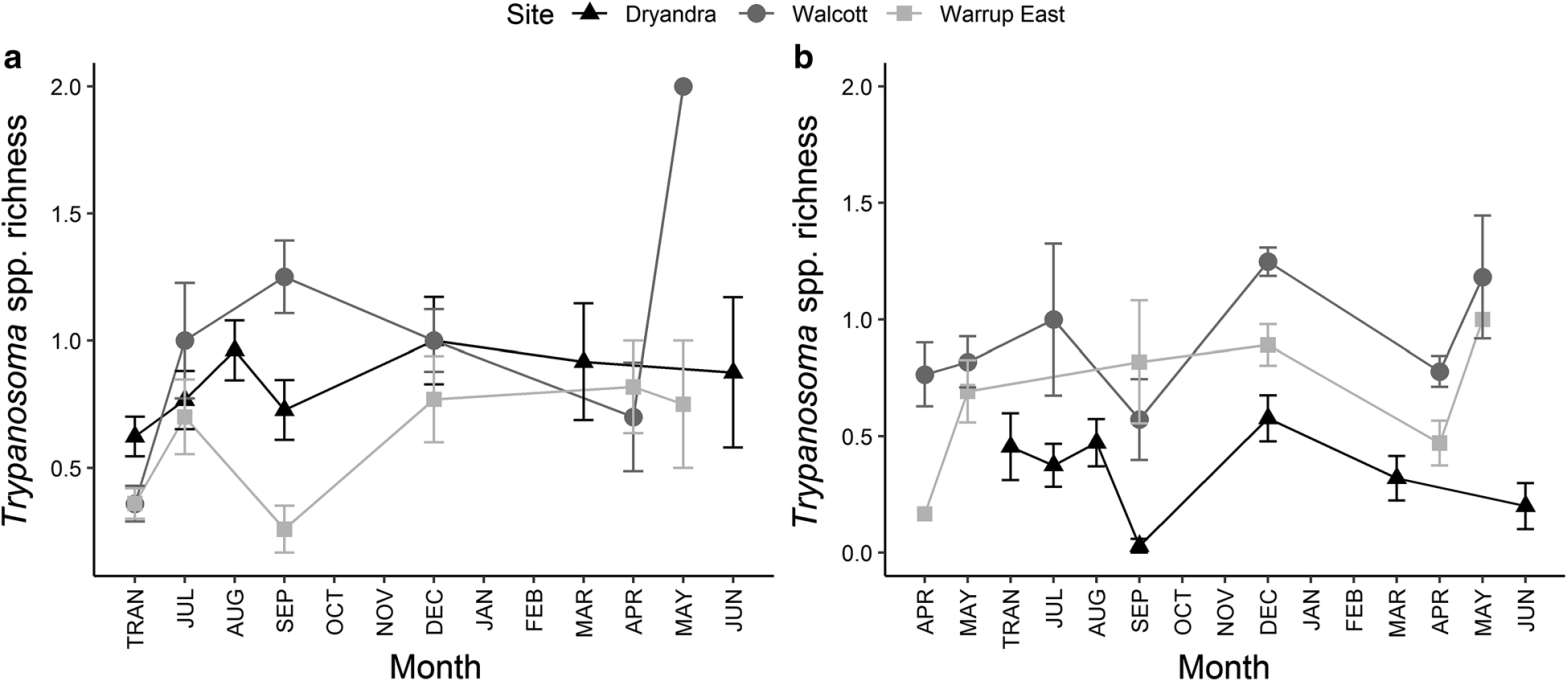
*Trypanosoma* spp. infracommunity richness (polyparasitism) over time (with standard error bars) in translocated (**a**) and resident (**b**) woylies. Error bars represent one standard error. TRAN: time of translocation

In translocated and resident woylies, *Trypanosoma* spp. richness differed significantly between sites (Table 1, Fig. 5); being highest (on average) within Walcott. In translocated woylies, *Trypanosoma* spp. richness was higher in Dryandra compared to Warrup East, while in resident woylies, Warrup East had a higher species richness than Dryandra. In *Trypanosoma*-positive woylies, *Trypanosoma* spp. polyparasitism was identified in 24% of cases. The maximum number of haemoparasites (including piroplasms) identified in a single host was four (Walcott translocated group; Table 2). In translocated woylies, *Trypanosoma* spp. richness increased with TST (Table 1, Fig. 5a) and the prevalence of haemoparasite co-infection increased from 40% to 63% following translocation. There was no effect of TST on *Trypanosoma* spp. richness in resident woylies (Table 1).

**Table 2:** The type and overall number of cases of haemoparasite co-infection identified in translocated and resident woylies from each site

	Dryandra	Walcott	Warrup East	Total
Translocated woylies				
*T. copemani*/*T. vegrandis*/*T. noyesi*	0	2	0	2
*T. copemani*/*T. vegrandis*	22	11	8	41
*T. copemani*/*T. noyesi*	0	5	3	8
*T. copemani*/*T.* ANU2	0	3	3	6
*T. vegrandis / T. gilletti*	0	0	5	5
*T. vegrandis/T. noyesi*	1	2	0	3
*T. noyesi*/*T.* ANU2	0	0	1	1
*Th. penicillata/T. copemani/T. vegrandis/T. noyesi*	0	1	0	1
*Th.* ANO2*/T. copemani/T. vegrandis/T. noyesi*	0	1	0	1
*Th. penicillata/T. copemani/T. vegrandis*	9	8	0	17
*Th. penicillata/T. copemani/T. noyesi*	0	3	2	5
*Th. penicillata/T. vegrandis/T. noyesi*	0	2	0	2
*Th.* ANO2*/T. copemani/T.* ANU2	0	1	1	2
*Th. penicillata/T. copemani*	41	3	8	52
*Th. penicillata/T. vegrandis*	13	13	0	26
*Th. penicillata/T. noyesi*	4	3	7	14
*Th. penicillata/T.* ANU2	1	1	1	3
*Babesia/T. copemani/T. vegrandis*	1	0	0	1
*Babesia/T. copemani*	5	0	0	5
*Babesia/T. noyesi*	1	0	0	1
*Th.* ANO2*/T. copemani*	1	1	2	4
*Th.* ANO2*/T. vegrandis*	0	0	1	1
*Th.* ANO2*/T. noyesi*	0	1	3	4
*Th.* ANO2*/T.* ANU2	0	0	1	1
Total	99	61	46	189
Resident woylies				
*T. copemani*/*T. vegrandis*/*T. noyesi*	0	3	0	3
*T. vegrandis*/*T. noyesi*/*T.* ANU2	0	1	0	1
*T. copemani*/*T. vegrandis*	9	50	6	65
*T. copemani*/*T. noyesi*	0	1	2	3
*T. copemani*/*T.* ANU2	0	1	0	1
*T. vegrandis/T. gilletti*	0	1	1	2
*T. vegrandis/T. noyesi*	0	5	2	7
*T. vegrandis*/*T.* ANU2	0	0	1	1
Total	9	62	12	83

**Fig. 6:**
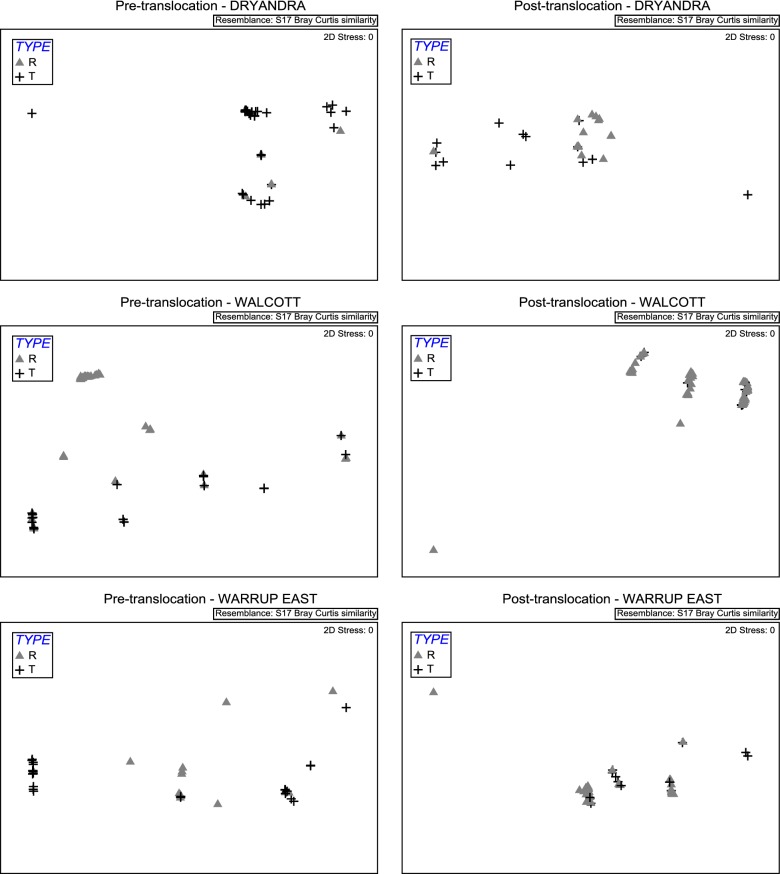
Non-metric multidimensional scaling plots of parasite communities in translocated (TYPE T) and resident (TYPE R) woylies pre-translocation (left; all time points prior to and including the point of translocation) and 6 months post-translocation (right)

**Fig. 7:**
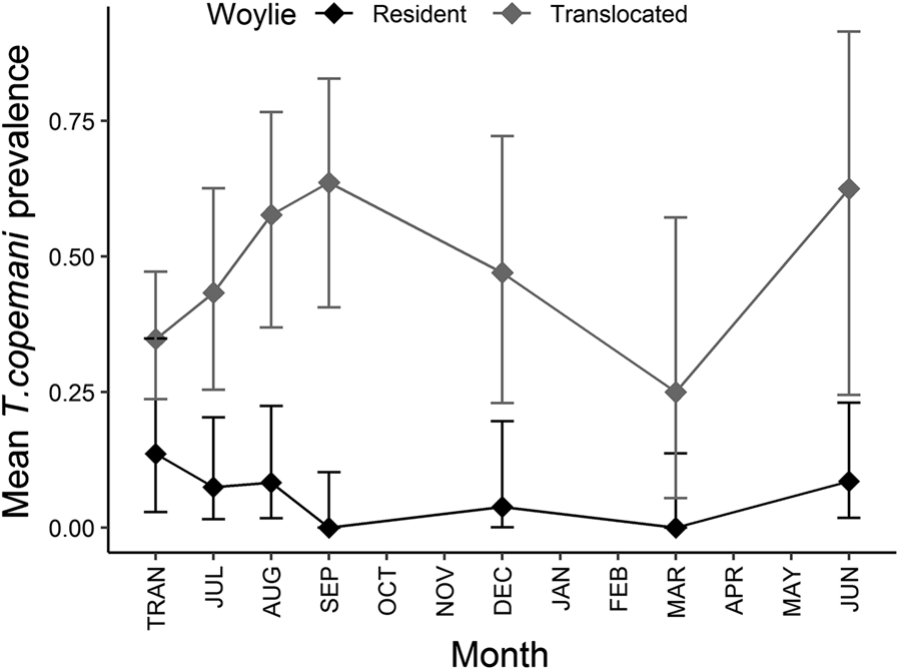
*Trypanosoma copemani* prevalence over time (with 95% CI) in translocated *versus* resident woylies within Dryandra. TRAN: time of translocation

Prior to translocation, *Trypanosoma* spp. community composition differed significantly between translocated and resident woylies in Walcott and Warrup East, but not Dryandra; this dissimilarity (84%) was mainly associated with differences in *T. vegrandis* prevalence (Additional file 1: Tables S3-S5). Six months after translocation, there was a significant difference between translocated and resident woylies within Dryandra, but not the Upper Warren sites (Fig. 6). This dissimilarity (61%) was largely attributed to the prevalence of *T. copemani*, which was significantly higher in translocated compared to resident woylies at all time points within Dryandra (Fig. 7).

### Effect of ivermectin treatment on haemoparasite prevalence and community composition

We did not detect a significant effect of ivermectin treatment on the prevalence of any haemoparasite species in translocated woylies, although there was a marginally significant interaction (*P* = 0.047) between ivermectin treatment and TST for *T. vegrandis* (Table 1), with *T. vegrandis* prevalence being lower in treated compared to untreated hosts from September onwards (Additional file 5: Figure S4). A significant interaction between ivermectin treatment and site was also found for *Trypanosoma* spp. polyparasitism (Table 1). *Trypanosoma* species richness was lower in treated compared to untreated woylies at all time points within Dryandra, whereas species richness was on average higher in treated woylies within Warrup East (Additional file 6: Figure S5).

## Discussion

During this study, the response of haemoparasites following translocation was highly site-specific, with major changes in haemoparasite prevalence occurring within the first few months after translocation. *Trypanosoma* spp. richness and the prevalence of haemoparasite co-infection increased following translocation. *Trypanosoma* spp. community composition became more similar over time within Walcott and Warrup East, but not Dryandra, where translocated woylies maintained a significantly higher prevalence of *T. copemani* infection compared to resident woylies. Ivermectin treatment had no significant effect on haemoparasite prevalence. We identified several new haemoparasites infecting woylies from the Upper Warren and Dryandra region. To our knowledge, this is the first published report of a *Bodo* sp. isolated from a marsupial blood sample; however *Bodo* sp. have been found previously in mammalian blood and tissue (Botero et al., unpublished data).

### Haemoparasite differences among sites

An important observation from this study is that haemoparasite prevalence, *Trypanosoma* spp. richness and *Trypanosoma* spp. community composition differed significantly between sites. Overall, Walcott was the most parasite-rich site, whilst Dryandra was comparatively parasite-poor; though translocated hosts within Dryandra maintained a high prevalence of haemoparasite infection (particularly *T. copemani*) following translocation. Differences in environmental conditions, woylie population density, contact with cohabiting species and the presence/absence of appropriate vectors may all have contributed towards the differences in haemoparasite prevalence and community composition and the site specific response of haemoparasites to translocation. As the translocations to the Upper Warren and Dryandra occurred 12 months apart, we may also be observing temporal fluctuations in parasite prevalence.

We also identified apparent site specificity of certain haemoparasites during this study. The absence of *Trypanosoma* sp. ANU2 in Dryandra resident woylies (and translocated hosts within Dryandra post-release) suggests that this trypanosome is specific to the Upper Warren region only. The absence of *Trypanosoma* sp. ANU2 in cohabiting marsupials (the brushtail possum *Trichosurus vulpecula hypoleucus* and the chuditch *Dasyurus geoffroii*) within Dryandra, but not the Upper Warren (Northover et al., unpublished data) supports this theory. Site differences in the prevalence of *T. gilletti*, *Th. apogeana* genotype ANO2 and *Babesia* were also observed, but difficult to interpret given the low prevalence of these parasites. Site differences in the type and prevalence of parasites (and their vectors) highlights the importance of parasite monitoring, particularly with regard to the inclusion of cohabiting host species within the release site, to gain a greater understanding of the parasites a translocated host might acquire (or potentially lose) following translocation. Likewise, incorporation of a control site that monitors host-parasite dynamics in the absence of translocation would be useful for identifying normal fluctuations in parasite prevalence, however may not be realistic when working with threatened host species.

### Changes in haemoparasite structure over time

In translocated woylies, significant changes in parasite prevalence occurred within the first few months after translocation, with *Trypanosoma* spp. richness and the prevalence of haemoparasite co-infection increasing. As follow-up monitoring of reintroduced populations commonly report parasite loss during translocation [10, 29, 30, 40], this outcome was unexpected; though supplementing pre-existing populations might increase the likelihood of parasite persistence particularly when resident conspecifics are infected with the same parasites. For haemoparasites infecting a host, it would seem inevitable that they will be translocated along with their host given their location in the blood stream; however, their persistence following translocation is usually dependent upon the presence of an appropriate arthropod vector and sufficient host density to enable parasite transmission. In the case of Walcott and Warrup East, it would appear that resident woylie density and the prevalence of infection (and vectors) within each site were sufficient to maintain, and even enhance, parasitaemia in translocated woylies after translocation; which resulted in *Trypanosoma* spp. community composition in translocated and resident woylie groups becoming more similar over time. Alternatively, enhanced parasitaemia may be the result of recrudescence of latent infections (e.g. secondary to stress), as has been reported for piroplasms in other species [41]. With the exception of *T. copemani* G2 (see below), however, there is no evidence to suggest that recrudescence of latent trypanosome infection occurs in woylies.

Within Dryandra, *Trypanosoma* spp. community composition diverged after translocation as translocated woylies maintained a significantly higher prevalence of *T. copemani* infection compared to residents. The introduction of translocated woylies with a much higher prevalence of *T. copemani* did not appear to affect infection prevalence in residents, which suggests that the prevalence of trypanosome vectors within Dryandra is low and/or contact with trypanosome-infected vectors/conspecifics occurs infrequently. Differences in *T. copemani* prevalence may be associated with the specific genotype of *T. copemani* infecting translocated and resident woylies. A high number of woylies infected with *T. copemani* G2 have been identified from the Upper Warren region [22], the origin of the translocated animals. Measurable parasitaemia associated with active *Trypanosoma* infection is typically transitory [42], but it has been proposed that *T. copemani* G2 is capable of invading tissues, replicating and sporadically re-entering the blood stream [18, 23, 43] in a similar manner to *Trypanosoma cruzi*. If this is the case and if *T. copemani* G2 was present in translocated woylies, this could explain the consistently high detection in these animals. Alternatively, translocation-associated stress may have promoted reactivation of infection, thereby increasing the number of trypomastigotes entering the blood (and the likelihood of detecting infection in the present study). Reactivation of chronic *T. cruzi* infection has been linked with immunosuppression in humans [44]. While a similarity to *T. cruzi* is a plausible explanation, a recent study looking into the proposed intracellular life-cycle of *T. copemani* showed no evidence that *T. copemani* G2 can actually replicate in cells [45]. However, there is clearly an interaction between *T. copemani* G2 and the host that is not entirely understood. *Trypanosoma copemani* infection seems to be more persistent than other *Trypanosoma* infections [18–20] and ultimately leads to a decrease in cell health *in vitro* [45]. In animals that are stressed this interaction could make parasite infection more difficult to eliminate.

### The efficacy of ivermectin treatment

The significant interaction between ivermectin treatment and site for *Trypanosoma* spp. polyparasitism was surprising. However, contrasting effects in different sites limits our ability to interpret the biological significance of this result. Likewise, the importance of the interaction between ivermectin treatment and TST for *T. vegrandis* is unclear, given the small effect we observed. If treatment-induced changes in *Trypanosoma* spp. polyparasitism and *T. vegrandis* prevalence are biologically significant however, this could have important consequences for host health given the association between *T. copemani*, *Trypanosoma* spp. co-infection, and declining woylie populations [17–20]; particularly if *T. vegrandis* imposes some form of competitive dominance over *T. copemani*, as previous studies have suggested [19, 46]. Further manipulative experiments that evaluate the effect of ivermectin on trypanosomes, and arthropod vectors, may be warranted if ivermectin is to be used in future woylie translocations. Likewise, studies that examine changes in the haemoparasite community in conjunction with measures of host health would assist with determining the biological significance of haemoparasite infection in woylies.

### Novel haemoparasite species

Lastly, we identified three novel haemoparasites infecting woylies. *Trypanosoma* sp. ANU2 was first isolated from woylies during this study, and has been phylogenetically characterised by Cooper et al. [22]. The *Babesia* sp. we identified (*Babesia* sp. 28, JQ682873) has been formerly described in woylies [25], but was reported to be most similar (98.4%; using a 527 bp alignment) to *Babesia occultans*, the causative agent of cattle babesiosis [47]. Our results (using a larger 693 bp alignment) suggest that this *Babesia* sp. is most similar to other marsupial-derived species. This is the first report of *Th. apogeana* genotype ANO2 in woylies, and this piroplasm is most similar to the recently described *Th. apogeana* from *Ixodes tasmani* on a dog in Tasmania [48]. Previous phylogenetic analysis [48] clustered *Th. apogeana* within a clade of *Theileria* spp. found in Australian marsupials, which was also the case here. While the vector of *Th. apogeana* genotype ANO2 in woylies is unknown, *Ixodes australiensis* and *Ixodes myrmecobii* were the only species of tick identified on woylies infected with *Th. apogeana* genotype ANO2 at the time of, or in the month preceding sample collection (Northover et al., unpublished data).

The detection of *Bodo* sp. DNA in the blood of a woylie was surprising given that the group is comprised of free-living species. However, environmental contamination cannot be ruled out, in a similar way to how *Bodo* sp. DNA was discovered in bats [49]. Woylies are ground dwelling herbivores feeding mostly on underground fungi [14], an environment in which *Bodo* sp. thrive. Thus, the potential transfer of DNA through the intestinal mucosa into the host’s blood circulation could be a likely consequence. However, the theory of trypanosomes evolving from free-living *Bodo* spp. [49] also needs to be considered.

### Future considerations for evaluating co-infection

It is worth noting that our molecular screening technique may have limited our ability to accurately detect mixed infections, leading to an underestimate of the extent of polyparasitism in our study. For example, species-specific trypanosome primers may identify the *Trypanosoma* species with the greatest parasitaemia. During this study, intermittent detection of haemoparasites was common and we often observed changes in the predominant species of haemoparasite and the presence/absence of co-infection. For parasites that can only be identified *via* DNA sequencing (e.g. *T. gilletti*) it is likely that their true prevalence is under-estimated. The use of alternative screening techniques such as targeted amplicon next generation sequencing (NGS), which can be superior for detecting co-infection [22] could be used to examine polyparasitism within a host and benefit translocation planning through the identification of potentially harmful *Trypanosoma* genotypes (e.g. *T. copemani* G2) within certain populations. Given the potential role of trypanosomes in the recent decline of the woylie, which resulted in the conservation status of this species being elevated to critically endangered, NGS technology may be more adept for monitoring perturbations to the parasite community following translocation in greater detail, thus enabling more informed decisions for the management of woylies and their parasite taxa during future translocations. Despite this limitation, we still detected a relatively high (40–63%) instance of polyparasitism in our study.

## Conclusions

As fauna translocations form an integral part of fauna conservation and management, consideration needs to be given to the biological implications of altering the parasite community within a host. To our knowledge, this is the first study to evaluate haemoparasite prevalence and composition in translocated and resident animals following translocation. The results from our study suggest that major changes in the host-parasite community happen relatively quickly (within the first 3 months). The unexpected observation that *Trypanosoma* spp. richness and the prevalence of haemoparasite co-infection increased after translocation, suggests that the outcome of fauna supplementations may differ from reintroductions where parasite loss typically occurs. Our study adds further weight to the idea that the response of haemoparasites following translocation can be highly site-specific and further understanding of what site characteristics drive these responses would improve our ability to predict how parasites may respond to translocation. Future studies that examine haemoparasites infecting subsequent generations of woylies would be useful for identifying population level haemoparasite changes, and separating within-individual effects (e.g. prevalence fluctuating with factors such as age, stress, condition, breeding status, season) from translocation-associated effects, which we were unable to do here. Importantly, the value of long-term parasite monitoring and undertaking well-designed scientific studies that examine parasite dynamics following experimental manipulation cannot be over-emphasised, particularly with regard to the collection of comprehensive mark-recapture data that can detect changes in the parasite community over time. Lastly, consideration needs to be given to the effects of antiparasitic drug treatment, which as observed here may be indirect, with potentially adverse consequences for host health. Studies that utilise next generation sequencing rather than traditional PCR assays and Sanger sequencing will be more adept at accurately quantifying changes in the parasite infracommunity during translocation.

## Additional files

**Additional file 1: Table S1.** Generic and species-specific primers used for the detection of haemoparasites in woylies. **Table S2.** Molecular methods for piroplasm PCR and Sanger sequencing/phylogenetic analyses of haemoparasites. **Table S3.** Number of woylies sampled within Dryandra, with prevalence of infection (and Jeffrey’s 95% CI). **Table S4.** Number of woylies sampled within Walcott, with prevalence of infection (and Jeffrey’s 95% CI). **Table S5.** Number of woylies sampled within Warrup East, with prevalence of infection (and Jeffrey’s 95% CI). **Table S6.** Genetic similarity between haemoparasites isolated during this study, and other known species.

**Additional file 2: Figure S1.** Phylogenetic relationship of *Trypanosoma* sp. ANU2 MF459625 compared to other trypanosomes, inferred using maximum likelihood analysis. We have included 30 sequences from GenBank, including three outgroups (*Bodo saltans*, *Bodo curvifilus* and *Trypanosoma brucei rhodesiense*), to validate the 1371 bp *18S* rDNA alignment.

**Additional file 3: Figure S2.** Phylogenetic relationship of *Theileria apogeana* genotype ANO2 MK182522 (and *Babesia* sp. 28 JQ682873) compared to other piroplasms, inferred using the Bayesian method. Fifty nine sequences from GenBank, plus one outgroup sequence (*Plasmodium falciparum* M19171), have been included to validate the 693 bp *18S* rDNA alignment.

**Additional file 4: Figure S3.** Phylogenetic relationship of *Bodo* sp. ANO4 MK182523 compared to other bodonida species, inferred using the maximum likelihood analysis. Eleven sequences from GenBank, plus one outgroup sequence (AJ009142), have been included to validate the 725 bp *18S* rDNA alignment.

**Additional file 5: Figure S4.** *Trypanosoma vegrandis* prevalence (all sites combined) over time (with 95% CI) in treated *versus* untreated translocated woylies (TRAN: time of translocation).

**Additional file 6: Figure S5.** *Trypanosoma* spp. polyparasitism over time (with standard error bars representing 1 SE) in treated *versus* untreated translocated woylies in (**a**) Dryandra and (**b**) Warrup East (TRAN: time of translocation).
