# Supplementary material for: Increased Trypanosoma spp. richness and prevalence of haemoparasite co-infection following translocation
Source: Parasit Vectors. 2019 Mar 21;12:126. doi: 10.1186/s13071-019-3370-6 (PMC6427866; doi:10.1186/s13071-019-3370-6)
Supplement: Supplementary file 1 — Additional file 1: Table S1. Generic and species-specific primers used for the detection of haemoparasites in woylies. Table S2. Molecular methods for piroplasm PCR and Sanger sequencing/phylogenetic analyses of haemoparasites. Table S3. Number of woylies sampled within Dryandra, with prevalence of infection (and Jeffrey’s 95% CI). Table S4. Number of woylies sampled within Walcott, with prevalence of infection (and Jeffrey’s 95% CI). Table S5. Number of woylies sampled within Warrup East, with prevalence of infection (and Jeffrey’s 95% CI). Table S6. Genetic similarity between haemoparasites isolated during this study, and other known species. [file 13071_2019_3370_MOESM1_ESM.docx]

**Additional file 1: Table S1.** Generic and species-specific primers used for the detection of haemoparasites in woylies.

|  |  | **External primer (5’-3’)** | **Annealing** | **Internal primer (5’-3’)** | **Annealing** | **Band** |
| --- | --- | --- | --- | --- | --- | --- |
|  |  |  | **temperature** |  | **temperature** | **size** |
| **Nested PCR** | |  |  |  |  |  |
| Generic Trypanosome | | SLF | 55°C | S825F | 56°C | 959bp |
|  |  | GCTTGTTTCAAGGACTTAGC |  | ACCGTTTCGGCTTTTGTTGG |  |  |
|  |  | S762R |  | SLIR |  |  |
|  |  | GACTTTTGCTTCCTCTAATG |  | ACATTGTAGTGCGCGTGTC |  |  |
| Generic Piroplasm | | BTF1 | 58°C | BTF2 | 62°C | 800bp |
|  |  | GGCTCATTACAACAGTTATAG |  | CCGTGCTAATTGTAGGGCTAATAC |  |  |
|  |  | BTR1 |  | BTR2 |  |  |
|  |  | CCCAAAGACTTTGATTTCTCTC |  | GGACTACGACGGTATCTGATCG |  |  |
| **Species-specific PCR** | |  |  |  |  |  |
| *Trypanosoma copemani* | | S825F | 56°C | WOF | 56°C | 457bp |
|  |  | ACCGTTTCGGCTTTTGTTGG |  | GTGTTGCTTTTTTGGTCTTCACG |  |  |
|  |  | SLIR |  | WOR |  |  |
|  |  | ACATTGTAGTGCGCGTGTC |  | CACAAAGGAGGAAAAAAGGGC |  |  |
| *Trypanosoma vegrandis* | | TVEF | 58°C | TVIF | 58°C | 350bp |
|  |  | GGGGTCCTTTTATTTTATTTG |  | GACCAAAAACGTGCACGTG |  |  |
|  |  | TVER |  | TVIR |  |  |
|  |  | TAATTTATTGGCCAGACAAA |  | AAATCGTCTCCGCTTTAAC |  |  |
| *Trypanosoma noyesi* | | H25EF | 58°C | H25IF | 62°C | 400bp |
|  |  | GCCGACAGTGCATTTTGT |  | TTTGAGGCGCAATGGTTTAG |  |  |
|  |  | H25ER |  | H25IR |  |  |
|  |  | GAGCGAGATGAACTCGACC |  | CGAGTTGAGGGAAGGTGGC |  |  |

**Additional file 1:** **Table S2.** Molecular methods for piroplasm PCR and Sanger sequencing/phylogenetic analyses of haemoparasites.

|  |  |  |
| --- | --- | --- |
|  | **PCR protocol - piroplasms** (modified from [34]) |  |
|  | A 24 µl master mix was made up of 0.8 µM of each primer (forward and reverse; see Additional file 1: Table S1), 2 mM MgCl_2_, 200 µM dNTPs and 0.2 U of Taq Pol, with the subsequent addition of 2 µl of DNA template. The cycling conditions consisted of a pre-PCR step of 94°C for 3 min, 58°C for 1 min and 72°C for 2 min, followed by 35 cycles of 94°C for 30 sec, 58°C for 20 sec, and 72°C for 30 sec, with a final extension temperature of 72°C for 7 min. |  |
|  |  |  |
|  | **Sanger sequencing protocol** |  |
|  | Purified amplicons were sequenced in both directions using an ABI Prism^TM^ Terminator Cycle Sequencing Kit on an Applied Bio-systems 3730 DNA Analyser (Applied Bio-systems, California, USA). To control for GC rich regions in the samples, the denaturation stage of the sequencing PCR was extended from 2 min to 10 min, and a 3:1 ratio of BigDye Terminator v3.1 Ready Reaction mix and dGTP BigDye Terminator v3.0 Ready Reaction mix was used (as opposed to just BigDye Terminator v3.1) in order to generate high quality chromatograms. Sequence identity for each sample was confirmed by using MUSCLE [50] to align each sequence against reference libraries downloaded from GenBank using Geneious v 8.1. |  |
|  |  |  |
|  | **Phylogenetic analyses** |  |
|  | The construction of each tree was dependent on the substitution model selected by jModeltest [51]. All trees were generated used a GTR + G + I substitution model. For *Trypanosoma* sp. ANU2 and *Bodo* sp. ANO4, bootstrap support for 1000 replicates was performed for maximum likelihood analysis using MEGA v.7 [52]. The Bayesian method (10,000,000 generations, burn- in 3,000 and sampling frequency of 1,000) was selected to construct a phylogenetic tree for *Theileria apogeana* genotype ANO2. The tree was run using Mr Bayes v. 3.1.2 [53]. |  |
|  |  |  |

**Additional file 1: Table S3.** Number of woylies sampled within Dryandra, with prevalence of infection (and Jeffrey’s 95% CI).

**Additional file 1:** **Table S4.** Number of woylies sampled within Walcott, with prevalence of infection (and Jeffrey’s 95% CI).

**Additional file 1: Table S5.** Number of woylies sampled within Warrup East, with prevalence of infection (and Jeffrey’s 95% CI).

**Additional file 1:** **Table S6.** Genetic similarity between haemoparasites isolated during this study and other known species.

| **Haemoparasite species** | **GenBank ID** | **Similarity** | **Source/Host species** | **Reference** |
| --- | --- | --- | --- | --- |
|  |  |  |  |  |
| *Trypanosoma* sp. ANU2 | MF459652 |  |  |  |
| *Trypanosoma copemani* G1 | KC753530 | 91.2% | Woylie, *Bettongia Penicillata* | [18] |
| *Trypanosoma copemani* G2 | KC753531 | 91.1% | Woylie, *Bettongia Penicillata* | [18] |
| *Trypanosoma copemani* Charlton | GU966588 | 91.1% | Koala, *Phascolarctos cinereus* | [33] |
|  |  |  |  |  |
| *Theileria apogeana* genotype ANO2 | MK182522 |  |  |  |
| *Theileria apogeana* | MG758116 | 96.7% | Tick, *Ixodes tasmani* | [48] |
| *Theileria brachyuri* PSC12 | DQ437685 | 94.1% | Quokka, *Setonix brachyurus* | [24] |
| *Theileria worthingtonorum* n. sp. ITF5 | MG758121 | 93.5% | Tick, *Ixodes tasmani* | [48] |
| *Theileria fuliginosus* | DQ437686 | 89.4% | Western grey kangaroo, *Macropus fuliginosus* | [24] |
|  |  |  |  |  |
| *Babesia* sp. 28 | JQ682873 |  |  |  |
| *Babesia* sp. ALT strain 2 | JQ437266 | 95.2% | Eastern grey kangaroo, *Macropus giganteus* | [54] |
| *Babesia* sp. voucher AB2015-P38 | KX361183 | 94.5% | Northern brown bandicoot, *Isoodon macrourus* | [55] |
| *Babesia occultans* | EU376017 | 92.7% | Sable antelope, *Hippotragus niger* | [56] |
|  |  |  |  |  |
| *Bodo* sp. ANO4 | MK182523 |  |  |  |
| *Bodo curvifilus* | AY425015 | 93.4% | Red deer (*Cervus elaphus*) faeces | [57] |
| *Bodo sorokini* strain ATCC | AY425018 | 86.1% | Russia (A.p. Mylnikov) | [57] |

**References**

1. Edgar RC. MUSCLE: multiple sequence alignment with high accuracy and high throughput. Nucleic Acids Res. 2004;32:1792–7.
2. Posada D. jModelTest: phylogenetic model averaging. Mol Biol Evol. 2008;25:1253–6.
3. Tamura K, Stecher G, Peterson D, Filipski A, Kumar S. MEGA6: Molecular Evolutionary Genetics Analysis version 6.0. Mol Biol Evol. 2013;30:2725–9.
4. Ronquist F, Huelsenbeck JP. MrBayes 3: Bayesian phylogenetic inference under mixed models. Bioinformatics. 2003;19:1572–4.
5. Dawood KE, Morgan JAT, Busfield F, Srivastava M, Fletcher TI, Sambono J, et al. Observation of a novel *Babesia* spp. in eastern grey kangaroos (*Macropus giganteus*) in Australia. Int J Parasitol Parasites Wildl. 2013;2:54–61.
6. Barbosa A, Reiss A, Jackson B, Warren K, Paparini A, Gillespie G, et al. Prevalence, genetic diversity and potential clinical impact of blood-borne and enteric protozoan parasites in native mammals from northern Australia. Vet Parasitol. 2017;238:94–105.
7. Oosthuizen MC, Zweygarth E, Collins NE, Troskie M, Penzhorn BL. Identification of a novel *Babesia* sp. from a sable antelope (*Hippotragus niger* Harris, 1838). J Clin Microbiol. 2008;46:2247–51.
8. Von der Heyden S, Chao EE, Vickerman K, Cavalier-Smith T. Ribosomal RNA phylogeny of bodonid and diplonemid flagellates and the evolution of Euglenozoa. J Eukaryot Microbiol. 2004;51:402–16.
