# Supplementary figures and images for: Increased Trypanosoma spp. richness and prevalence of haemoparasite co-infection following translocation
Source: Parasit Vectors. 2019 Mar 21;12:126. doi: 10.1186/s13071-019-3370-6 (PMC6427866; doi:10.1186/s13071-019-3370-6)

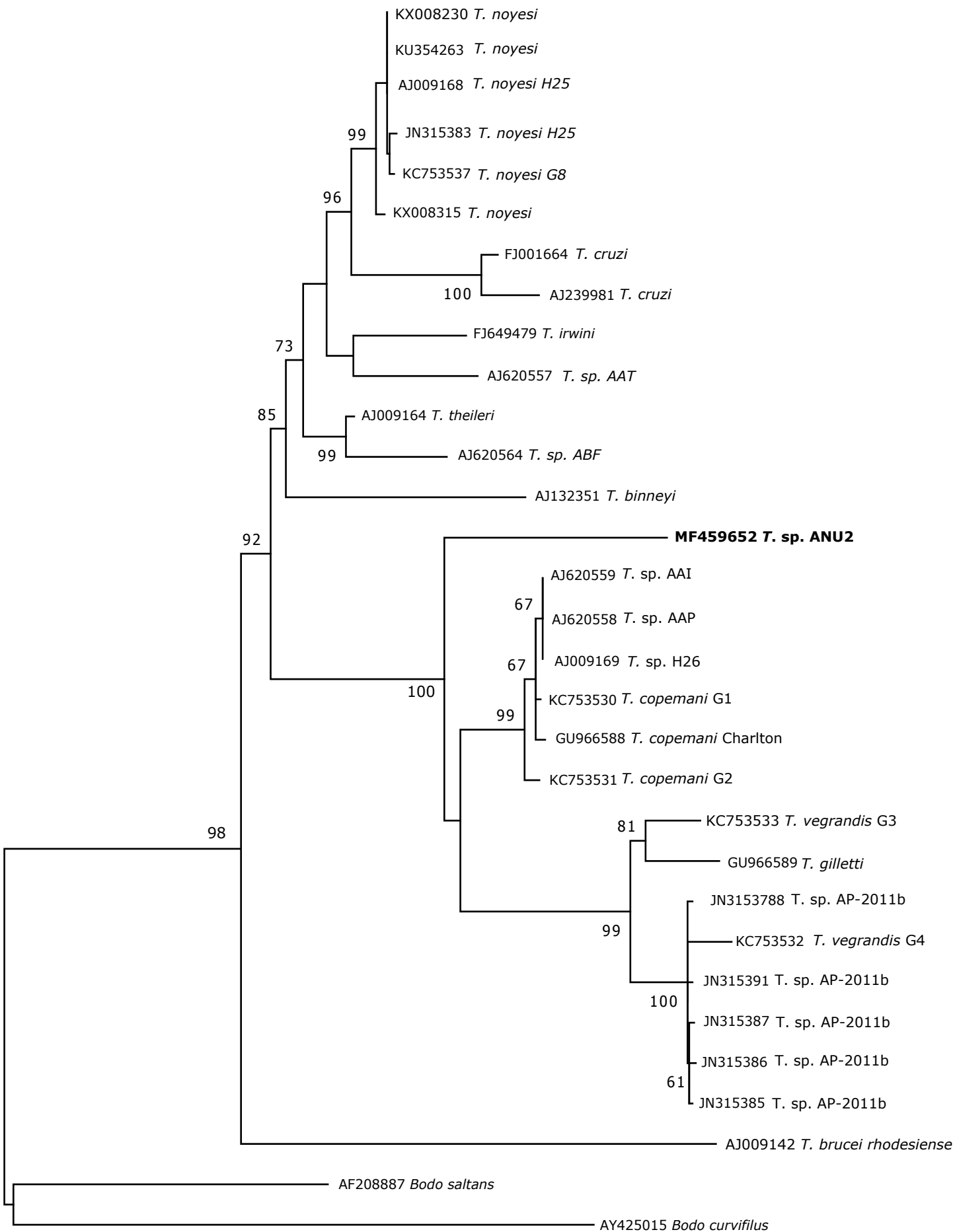

0.050

Supplement: Supplementary file 2 — Additional file 2: Figure S1. Phylogenetic relationship of Trypanosoma sp. ANU2 MF459625 compared to other trypanosomes, inferred using maximum likelihood analysis. We have included 30 sequences from GenBank, including three outgroups (Bodo saltans, Bodo curvifilus and Trypanosoma brucei rhodesiense), to validate the 1371 bp 18S rDNA alignment. [file 13071_2019_3370_MOESM2_ESM.pdf]

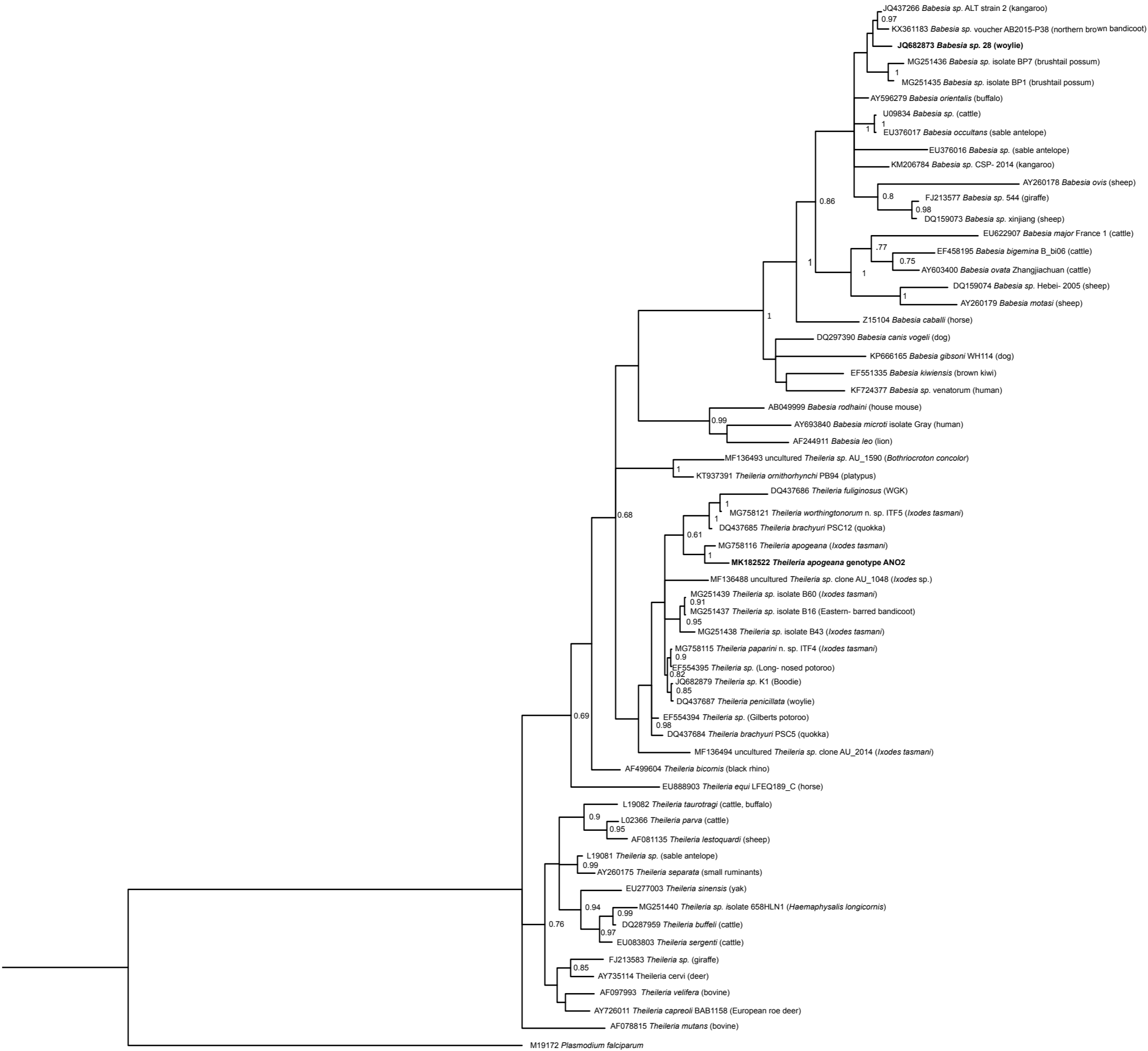

Supplement: Supplementary file 3 — Additional file 3: Figure S2. Phylogenetic relationship of Theileria apogeana genotype ANO2 MK182522 (and Babesia sp. 28 JQ682873) compared to other piroplasms, inferred using the Bayesian method. Fifty nine sequences from GenBank, plus one outgroup sequence (Plasmodium falciparum M19171), have been included to validate the 693 bp 18S rDNA alignment. [file 13071_2019_3370_MOESM3_ESM.pdf]

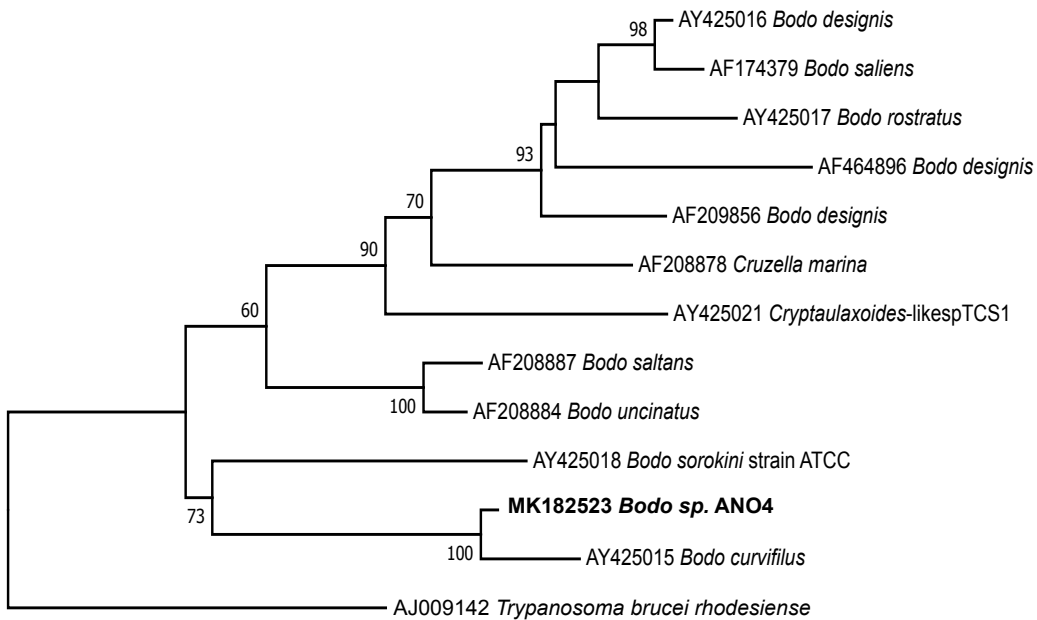

Supplement: Supplementary file 4 — Additional file 4: Figure S3. Phylogenetic relationship of Bodo sp. ANO4 MK182523 compared to other bodonida species, inferred using the maximum likelihood analysis. Eleven sequences from GenBank, plus one outgroup sequence (AJ009142), have been included to validate the 725 bp 18S rDNA alignment. [file 13071_2019_3370_MOESM4_ESM.pdf]

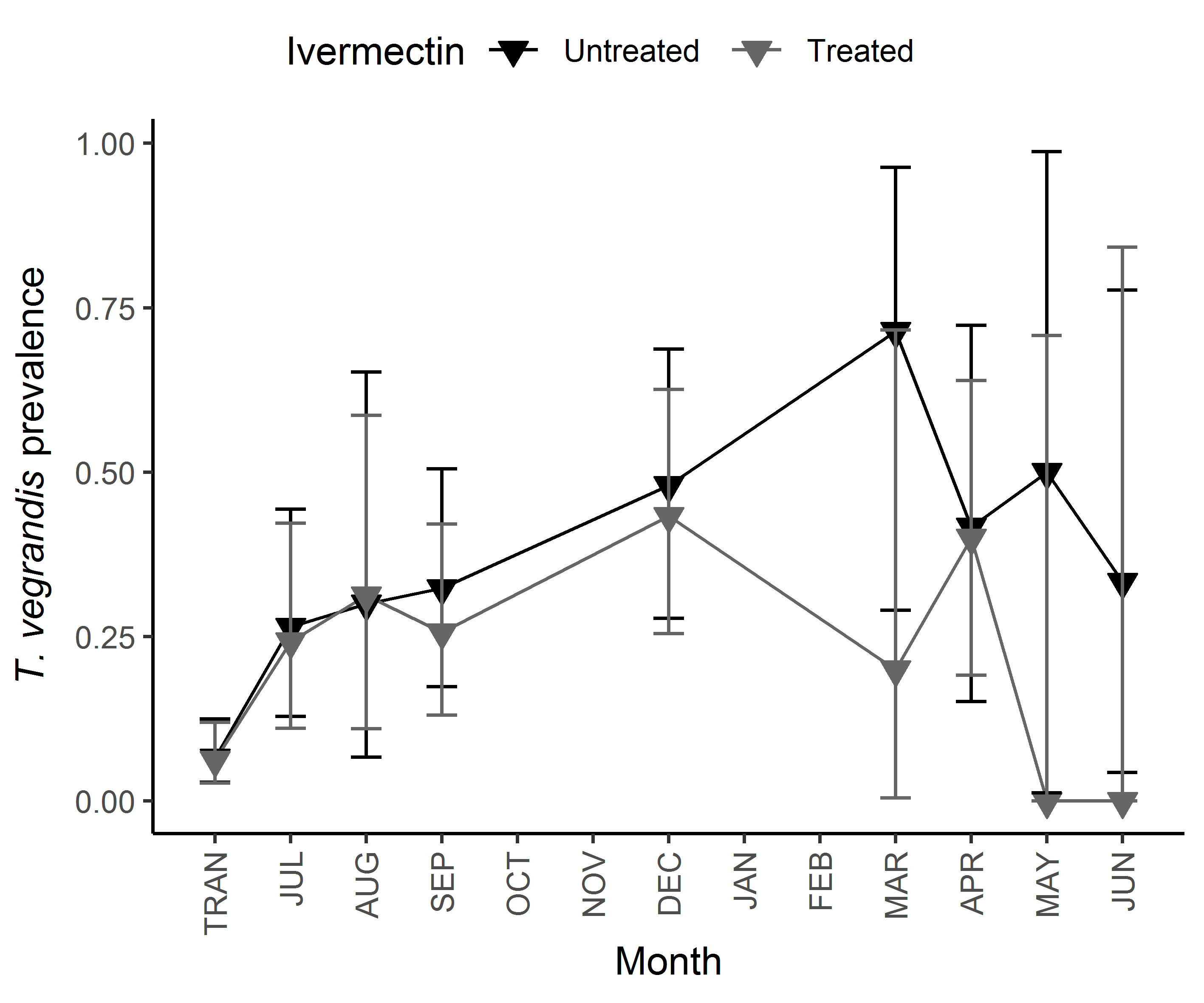

Supplement: Supplementary file 5 — Additional file 5: Figure S4. Trypanosoma vegrandis prevalence (all sites combined) over time (with 95% CI) in treated versus untreated translocated woylies (TRAN: time of translocation). [file 13071_2019_3370_MOESM5_ESM.tiff]

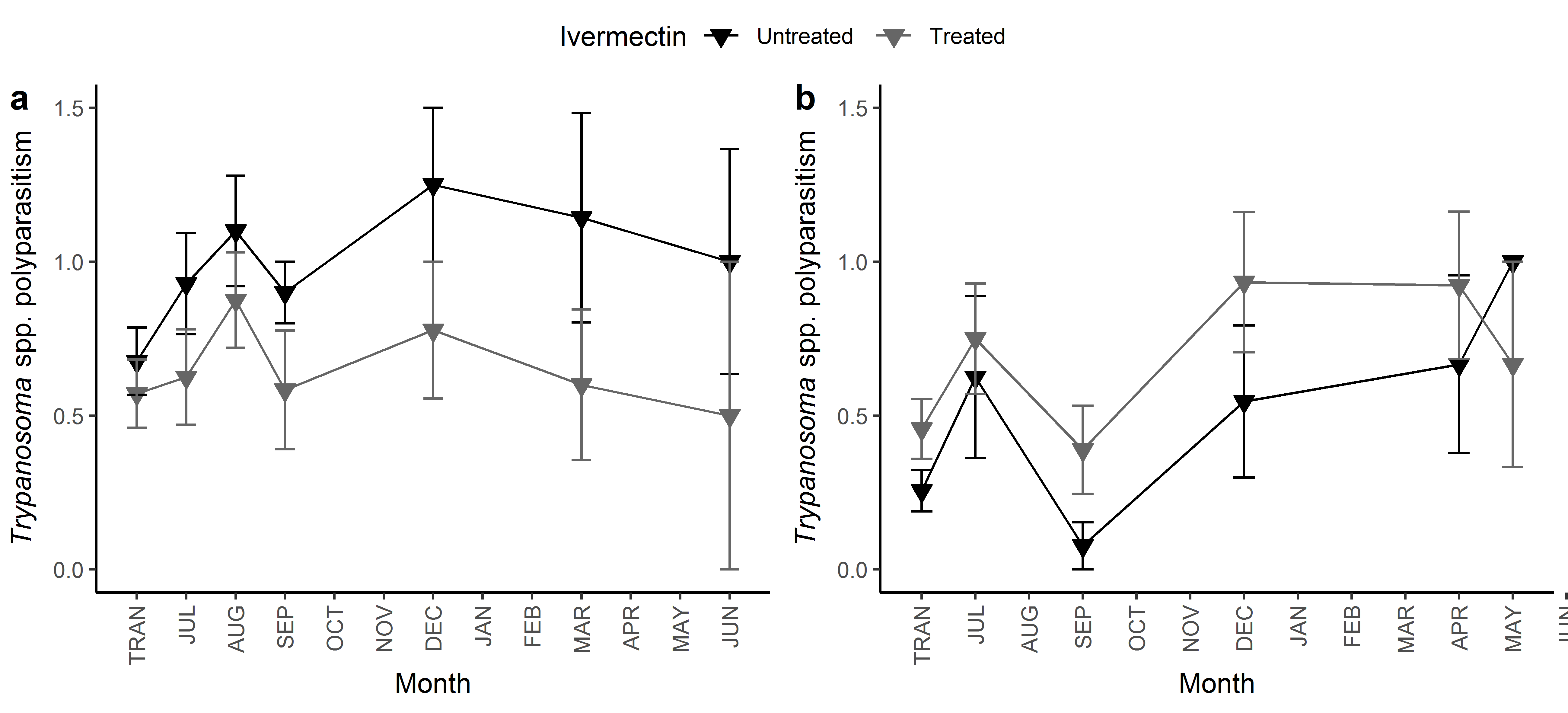

Supplement: Supplementary file 6 — Additional file 6: Figure S5. Trypanosoma spp. polyparasitism over time (with standard error bars representing 1 SE) in treated versus untreated translocated woylies in (a) Dryandra and (b) Warrup East (TRAN: time of translocation). [file 13071_2019_3370_MOESM6_ESM.tiff]
